# Mxi1 inhibits the proliferation of U87 glioma cells through down-regulation of *cyclin B1* gene expression

**DOI:** 10.1038/sj.bjc.6600065

**Published:** 2002-02-01

**Authors:** I Manni, P Tunici, N Cirenei, R Albarosa, B M Colombo, L Roz, A Sacchi, G Piaggio, G Finocchiaro

**Affiliations:** Istituto Regina Elena, Centro Ricerca Sperimentale, Laboratorio di Oncogenesi Molecolare, Via delle messi D'Oro 156, 00158 Roma, Italy; Istituto Nazionale Neurologico C. Besta, Unita' of Neuro-Oncologia e Terapia Genica, Via Celoria 11, 20133 Milano, Italy

**Keywords:** Mxi1, cyclin B1 promoter, tumour suppressor

## Abstract

Mxi1 is a Mad family member that plays a role in cell proliferation and differentiation. To test the role of Mxi1 on tumorigenesis of glioma cells we transfected a CMV-driven *MXI1* cDNA in U87 human glioblastoma cells. Two clones were isolated expressing *MXI1* levels 18- and 3.5-fold higher than wild-type U87 cells (clone U87.Mxi1.14 and U87.Mxi1.22, respectively). *In vivo*, U87.Mxi1.14 cells were not tumorigenic in nude mice and delayed development of tumours was observed with U87.Mxi1.22 cells. *In vitro*, the proliferation rate was partially and strongly inhibited in U87.Mxi1.22 and U87.Mxi1.14 cells respectively. The cell cycle analysis revealed a relevant accumulation of U87.Mxi1.14 cells in the G_2_/M phase. Interestingly, the expression of cyclin B1 was inhibited to about 60% in U87.Mxi1.14 cells. This inhibition occurs at the transcriptional level and depends, at least in part, on the E-box present on the *cyclin B1* promoter. Consistent with this, the endogenous Mxi1 binds this E-box *in vitro*. Thus, our findings indicate that Mxi1 can act as a tumour suppressor in human glioblastomas through a molecular mechanism involving the transcriptional down-regulation of *cyclin B1* gene expression.

*British Journal of Cancer* (2002) **86**, 477–484. DOI: 10.1038/sj/bjc/6600065 www.bjcancer.com

© 2002 The Cancer Research Campaign

## 

The oncogenic transformation by Myc proteins requires a basic helix-loop-helix leucine zipper (bHLH-zip)-mediated interaction with Max, the Myc obligate DNA-binding partner (Dang *et al*, 1989; Blackwell *et al*, 1990; Kretzner *et al*, 1992). Proteins of the Myc network are essential regulators of cell growth and differentiation (Henriksson and Luscher, 1996). The Mad family proteins (Ayer *et al*, 1993; Zervos *et al*, 1993; Hurlin *et al*, 1995), among which Mxi1, can antagonize Myc by interacting with Max. This complex recruits Sin3A or Sin3B transcriptional repressors, the co-repressor N-CoR, and the histone deacetylase HDAC1 (Schreiber-Agus *et al*, 1995; Rao *et al*, 1996; Alland *et al*, 1997; Hassig *et al*, 1997; Laherty *et al*, 1997). Direct repression of the *c-Myc* promoter by Mxi1 can also take place (Lee and Ziff, 1999).

Because of their molecular function, proteins of the Mad family are potentially involved in tumour suppression. In particular, a deficient function of these proteins could contribute to tumorigenesis by making available large amounts of Max for c-Myc activation. Chen *et al* (1995) have provided evidences for an inhibitory role of Mad1 on malignant gliomas. The recent knock out of *MXI1* in mice has confirmed its role as a tumour suppressor (Schreiber-Agus *et al*, 1998; Foley and Eisenman, 1999).

*MXI1* gene has been mapped to the chromosome region 10q25, frequently deleted in glioblastomas (Rasheed *et al*, 1992; Fults and Pedone, 1993; Edelhoff *et al*, 1994; Albarosa *et al*, 1996). The over-expression of Mxi1 in glioblastoma cells suppresses cell growth, inducing an accumulation of the cells in the G_2_/M phases of the cell cycle (Wechsler *et al*, 1997).

The target genes by which Mxi1 exerts its effect on the cell cycle progression are still not identified. Here we demonstrate that during the Mxi1-induced G_2_/M block in glioblastoma cells, the expression of the master regulatory gene of the G_2_ progression, *cyclin B1* is down-regulated at transcriptional level, indicating that cyclin B1 is a target of Mxi1 activity.

## MATERIALS AND METHODS

### Transfection of U87 cells by a Mxi1 eukaryotic expression plasmid

Total RNA was prepared after direct lysis of lymphocytes with Tripure reagent (Roche). Two rounds of reverse transcription were performed starting from 5 μg of total RNA, using oligo (dT) primer, other reagents and procedures contained in the cDNA Cycle Kit from Invitrogen. Four μl of the resulting cDNA, 50 pmoles of each primer, 0.2 mM dNTPs and 2.5 units of either Taq or HF-Taq DNA polymerase, with the respective buffers from Roche, were used for two rounds of PCR. The first round was performed using primers Mxi1-A1 (TAAGGGAGTGCGGAGAGG) and Mxi1-R (TTAAATACAGGTCCTCTGACCC). The initial denaturation at 94°C for 5 min was followed by 30 cycles at 94°C for 1 min, 55°C for 2 min and 72°C for 3 min. One μl of the PCR mixture was subjected to a second round of amplification under the same conditions using primers Mxi1-A1 and Mxi1-R2b (CATGCTGGGTTCTATGAAGAG). The resulting 740 bp fragment was cloned into pCR3 eukaryotic expression plasmid (Invitrogen) in both orientations. The sequence of amplified *MXI1* cDNA corresponds to wild-type *MXI1* except for two nucleotide changes at codon 178 (GAA to GGA) and 195 (AGT to GGT) predicting, respectively, a glycine for glutamate and a glycine for serine substitution. These changes, present in the plasmid transfected in the cells used for ^3^H incorporation assays, were likely due to PCR amplification, since they were never found by SSCP analysis of glioma and lymphocyte DNA of 36 unrelated individuals (G Finocchiaro unpublished data). Moreover they are not placed in the four regions that are critical for Mxi1 transcriptional activity (SR1 domain, basic region, HLH domain and leucine zipper domain, aa 1–147, ibidem). As assessed by sequence information, the cells used in the cell counting and colonies formation assays, have been transfected with a plasmid carrying a wild-type sequence of Mxi1 derived from a different PCR.

The human U87 glioma cell line (ATCC HTB 14), was grown in Eagle's medium (EMEM) supplemented with 10% foetal calf serum, non-essential amino acids, sodium pyruvate, L-glutamine, streptomycin and penicillin. U87 cells (80% confluent) were lipofected by DOTAP (Roche) using 5 μg of pCR3/Mxi1 purified by Qiagen tips 100. G418 selection was performed using 300 μg ml^−1^ of active drug (Sigma).

To evaluate MXI1 expression after transfection in U87 cells *MXI1* cDNA was amplified for 35 cycles with primers MXI1-A1 and MXI1-R2b (CATGCTGGGTTCTATGAAGAG) 10/50 μl of PCR mix were loaded. β-*actin* was amplified for 25 cycles with primers ß-Act-F2, ACCAACTGGGACGACATGGA and β-Act-R2, GTGGTGGTGAAGCTGTAGC and 5/50 μl of PCR mix loaded together with *MXI1* amplified from the same RNA. After agarose gel electrophoresis the amount of DNA loaded and the MXI1/actin ratio was evaluated using a Kodak DC40 camera and the Kodak Digital Science 1D software (Scientific Imaging System, New Haven, CT, USA).

### *In vitro* experiments

The proliferation assay was performed on wild-type, Mxi1.22 and Mxi1.14 clones. 5–10×10^3^ cells have been plated in quintuplicate in a 96 well culture plate. One μCi of [^3^H]-thymidine in 100 μl of culture medium (EMEM) was added to each well 3–4 h after seeding the cells. After 24 h a semi-automated cell harvesting apparatus was used to lyse cells with water and precipitate the labelled DNA on glass fibre filters. Filter pads were dried and counted in a liquid scintillation beta-counter. The proliferation rate was calculated as fold increases over the value obtained on day 1. For the cell counting assay the cells have been transfected with a plasmid carrying a wild-type Mxi1 cDNA both in sense or antisense orientations. After a selection with 300 μg ml^−1^ of G418 for 2 weeks 2×10^3^ were plated in triplicate in a 24-well culture plate. Cell counting was performed for the subsequent 4 days by Trypan blue staining. To test colony-forming ability 2×10^2^ transfected cells, coming from the same selection as above, were plated in triplicate in a 100 mM culture plate. After 3 weeks the medium was removed and colonies were stained with methylene blue 0.06% and glutaraldehyde 1.25% in Hanks' Balanced Salt Solution.

### *In vivo* experiments

Three groups of athymic ‘nude’ mice (females, 20–25 g, Charles River) were used. Six mice were inoculated subcutaneously in one flank with U87 wild-type cells, six with U87.Mxi1.22 cells and 11 with U87.Mxi1.14 cells. All inoculations consisted of 5×10^5^ cells resuspended in 100 μl of PBS. The tumour size was defined by calculating the major diameters with a caliper. The major diameters were multiplied and values given in mm^2^.

### Cell cycle analysis

For each sample 10^4^ events were analyzed by an Epics cytofluorimeter (Coulter). The cells were stained by propidium iodide (0.1 mg ml^−1^) and RNase (150 U ml^−1^) was added after permeabilization in PBS with 0.2% Triton X. DNA content and cell cycle distribution were determined using a computer-assisted analysis.

### Northern blot analysis

Total RNAs were extracted by the guanidinium thiocynate/phenol procedure (Sambrook *et al*, 1989) from U87 wt, U87.Mxi1.22 and U87.Mxi1.14 cell lines. Aliquots (20 μg) of total RNA were separated on 1% formaldehyde-agarose gel at 50 V for 18 h. Nylon N^+^ (Qiabrane) filters for Northern analysis were prepared by capillary transfer. Filters were hybridized with the following probes: (i) 1400 bp fragment from human *cyclin B1* cDNA obtained by *Bam*HI/*Hind*III digestion of a pCMX plasmid carrying the entire cyclin B1 cDNA cloned into *Bam*HI/*Hind*III sites, (ii) 1200 bp fragment from human cyclin A cDNA obtained by *Bam*HI/*Hind*III digestion of a pCMX plasmid carrying the entire cyclin B1 cDNA cloned into *Bam*HI/*Hind*III sites, (iii) 740 bp fragment from human *MXI1* cDNA obtained by PCR on the pCR3 expression vector carrying *MXI1* cDNA, (iv) β-*actin* (Clontech Laboratories Inc., CA, USA). The hybridization were performed at 42°C in a buffer containing 50% formamide, washed to a final stringency of 0.5×SSC, 0.1% SDS at 65°C and autoradiographed at −80°C. Densitometric analysis of autoradigrams were performed by the Molecular Analyst program (BioRad, CA, USA).

### Western blot analysis

Total-cell lysates were loaded and separated by SDS–PAGE (12% polyacrylamide) gel and electroblotted onto nitrocellulose. After staining in 0.2% Ponceau S in 3% TCA, the filter was washed twice in PBS and protein binding sites blocked in 5% non-fat dried milk in PBS. The filter was treated with (i) anti cyclin B1 mouse monoclonal (33 ng ml^−1^), (ii) cyclin A rabbit polyclonal (33 ng ml^−1^), (iii) Mxi1 rabbit polyclonal (33 ng ml^−1^), Hsp70 mouse monoclonal (33 ng ml^−1^) antibodies (Santa Cruz Biotechnology, Inc. CA, USA). After four washes in TBS (150 mM NaCl, 50 mM Tris-HCl, pH 7.9) the filter was incubated with the secondary antibody (anti-mouse Ig) conjugated with peroxidase, in 3% BSA/TBS for 1 h at room temperature. The filter was washed four times as above and Western blots were developed using the ECL procedure (Roche, Little Chalfont, UK).

### Transient transfections and CAT assay

U87, U87.Mxi1.22 and U87.Mxi1.14 cell lines were cultured in Eagle's medium (EMEM) supplemented with 10% foetal calf serum. DNA transfections were performed using calcium phosphate precipitation (Graham and Van Der Eb, 1973). In each 60 mm plate, 1.5×10^5^ cells were transfected with aliquots of precipitates containing 5 μg of p332B1CAT (Piaggio *et al*, 1995) or pmE-box332B1CAT (Farina *et al*, 1996) and 0.5 μg of cytomegalovirus-β-galactosidase (CMV-βgal) plasmid, a control for transfection efficiency. After 16 h, cells were washed with phosphate-buffered saline (PBS) and fresh medium was added. Cells were harvested 48 h after transfection and CAT activity was assayed in whole-cell extract as described (Desvergne *et al*, 1991). The values were normalized against β-galactosidase activity and protein contents of the extracts.

### Nuclear extracts and electromobility shift assays

Nuclear extracts from U87 cells were performed as described by Dignam *et al* (1983). The lysis was performed in the presence of the following protease and phosphatase inhibitors: leupeptin 10 μg ml^−1^, pepstatin 4 μg ml^−1^, aprotinin 5 μg ml^−1^, 50 mM NaF, 1 mM sodium orthovanadate. Electromobility shift assays were performed as described by Miltenberger *et al* (1995) with the following modifications: (i) oligonucleotide was labelled using the Klenow fragment of DNA polymerase I, (ii) the reaction was carried out on ice for 30 min and run was performed in 0.5×TBE buffer. In supershift experiments were used 100 ng of anti-Max rabbit polyclonal antibody, 2 μg of anti-Mxi1 rabbit polyclonal antibody, and 2 μg of anti-Mad2 goat polyclonal antibody (all provided by Santa Cruz Biotechnology). The following oligonucleotides were used as probe and competitor (consensus sites are underlined): B1 E-box 5′-GGGAGGCAGACCACGTGAGAGCCTGG; B1up CCAAT: 5′-CCGCAGCCGCCAATGGGAAGGGAGTGA (Farina *et al*, 1999).

## RESULTS

### Development of glioblastoma cell lines overexpressing* MXI1* cDNA and analysis of their proliferation rate

U87 cells, a cell line derived from a human glioblastoma, were stably transfected with a construct carrying *MXI1* cDNA. Human *MXI1* cDNA was amplified by RT–PCR on lymphocyte RNA and inserted in the eukaryotic expression plasmid pCR3, under the control of the CMV promoter. *MXI1* cDNA was also cloned in the antisense orientation, as verified by restriction site analysis and this construct was used for control experiments. Liposome-mediated transfer was used to transfect sense or anti-sense *MXI1* cDNAs into U87 cells. Cells transfected by *MXI1* cDNA had a decreased [^3^H]-thymidine incorporation compared to the controls, while cells transfected with antisense cDNA behaved like controls. After 7–8 weeks in culture, however, this negative effect on U87 proliferation decreased significantly (compare data obtained 1 and 2 months after transfection in Table 1

**Table 1:**
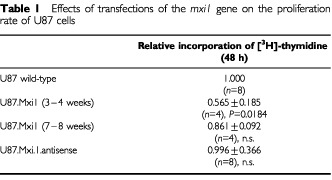
Effects of transfections of the *mxi1* gene on the proliferation rate of U87 cells

). To confirm our observation on a more stable population of transfected cells, 26 clones have been isolated by limiting dilution. Two of these clones showed, respectively, high and low-intermediate levels of *MXI1* expression (Figure 1

**Figure 1:**
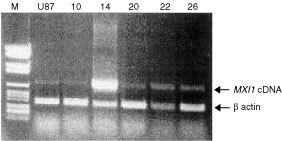
RT–PCR of *MXI1* and ß-*actin* cDNAs in U87 cells wild-type (second lane from left) and transfected by *MXI1* cDNA (clones 10, 14, 20, 22 and 26). Molecular weight markers (number VI, Roche) are in the first lane from left. Clones 14 (high *MXI1* expression) and 22 (low-intermediate *MXI1* expression) were employed in further experiments.

). Clone U87.Mxi1.14 had a *MXI1/actin* ratio, evaluated by densitometry of RT–PCR products, 18-fold higher than wild-type U87 cells (high level of expression). Clone U87.Mxi1.22 had a *MXI1/actin* ratio 3.5-fold higher than control (low-intermediate level of expression). The results of [^3^H]-thymidine incorporation of wild-type and *MXI1*-transfected U87 clones confirmed that increased Mxi1 expression causes a decreased proliferation rate of glioma cells at different time points. The incorporation rate of U87 wild-type cells, 1 week after seeding, was 2.6 times higher than U87.Mxi1.22 and seven times higher than U87.Mxi1.14, respectively, thus indicating a correlation between the levels of Mxi1 expression and the degree of growth inhibition (Figure 2A

**Figure 2:**
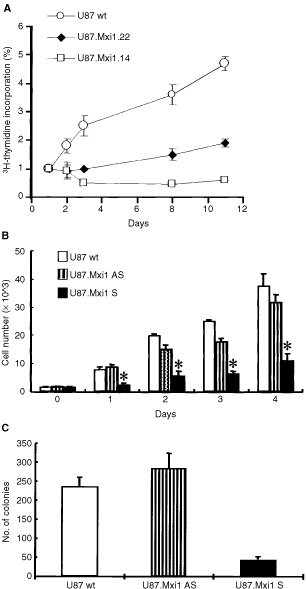
MXI1 decreases the proliferation rate *in vitro* of U87 cells. (**A**) [^3^H]-thymidine incorporation in U87 wild-type and in *Mxi1*-transfected clones 22 and 14, with low and intermediate high levels, respectively, of *Mxi1* expression. Data are the results of two experiments, and each point was evaluated five times. Values are shown as the folds of increase of [^3^H]-thymidine incorporation, over day 1. Standard errors are indicated. (**B**) Cells were transfected with pCR/Mxi1 as reported in Materials and Methods. On day 0, 2×10^3^ cells were plated in triplicate in a 24-wells culture plate and counted after 1, 2, 3 and 4 days. Each point represents the mean±s.d. of three independent experiments. *P<0.05 (Student *t*-test). (**C**) 2×10^2^ cells were plated in triplicate in a 100 mm culture plate. After 3 weeks cells were stained and counted as reported in Materials and Methods. Each point represents the mean±s.d. of three independent experiments.

).

Cell counting and colony forming ability were performed to evaluate the different proliferation rate of cells untransfected or transfected with pCR3/Mxi1 both in sense and antisense orientations. Results are reported in Figure 2B,C and confirm the significant decrease of the proliferation rate of glioma cells overexpressing *MXI1*. The number of U87/MXI1sense cells 4 days after plating was 30% that of untransfected cells and 35% that of U87/MXI1antisense cells (*P*<0.05). The number of clones of U87/MXI1sense cells was 25% that of untransfected cells and 22% that of U87/MXI1antisense cells (*P*<0.02).

### Mxi1 over-expression inhibits tumorigenesis of U87 cells in nude mice

**Figure 3:**
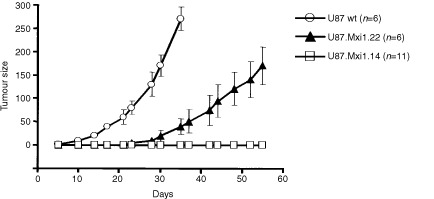
*In vivo* growth rate of U87 wild-type and *MXI1*-transfected cells (clone 22, intermediate *MXI1* expression; clone 14, high *MXI1* expression) after sub-cutaneous inoculation in nude mice. Tumour sizes, given in mm^2^, are calculated by multiplying tumour diameters. Standard errors are indicated. Animals with control tumours were sacrificed after 1 month because tumours were too large.

The effects of Mxi1 over-expression were studied *in vivo*, by subcutaneous grafting in athymic nude mice of U87 wild-type, U87.Mxi1.14 and U87.Mxi1.22 cells (Figure 3

). U87.Mxi1.22 tumours appeared later and their size was smaller than controls′ (*P*=0.0032 to *P*=0.0001 for comparable time points; *t*-test, two tails). Strikingly, mice injected with U87.Mxi1.14 cells did not develop any tumour for more than 1 month, after which only one animal showed the appearance of a neoplastic mass. One hundred and twenty days after tumour cell injection, when mice were sacrificed, 10 out of 11 were still tumour-free. These results are consistent with *in vitro* data, indicating that Mxi1 can act as a suppressor of U87 glioblastomas.

### U87 cells with high level of Mxi1 expression accumulate in the G_2_/M phase of the cell cycle

**Figure 4:**
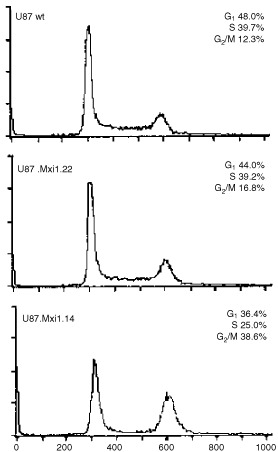
U87 cells with high level of Mxi1 expression accumulate in the G_2_/M phases of the cell cycle. FACS analysis shows the DNA content distribution of U87 wild-type, U87 Mxi1.14, and U87 Mxi1.22 cell lines. The percentages of cells in G_1_, S, and G_2_/M phases are indicated. X-axes represent the relative fluorescence intensity of propidium iodide-stained cells. Y-axes represent the cell number.

To determine whether Mxi1 inhibits cell proliferation inducing a block in a specific phase of the cell cycle, the DNA content of U87 wild-type, U87.Mxi1.14 and U87.Mxi1.22 cells was measured by flow cytometry. Figure 4

shows that 38.6% of U87.Mxi1.14 cells and 12.3% of wild-type cells, respectively, are in G_2_/M and that lower amounts of U87.Mxi1.14 cells are in G_1_ and S phases compared to the wild-type U87 (36.4% *vs* 48.0% and 25% *vs* 39.7% respectively). U87.Mxi1.22 cells, on the other hand, only show a little increase of cells in G_2_/M (16.8% *vs* 12.3% in wild-type cells), suggesting that Mxi1 effects are partially dose-dependent. These results demonstrate that Mxi1 over-expression in U87 glioblastoma cells leads to an accumulation in the G_2_/M phase of the cell cycle.

### Mxi1 inhibits expression of the *cyclin B1* gene

In normal cells, the B-type cyclins (B1, B2, and B3) control the G_2_/M transition of the cell cycle (Pines and Hunter, 1989; Glotzer *et al*, 1991). Cyclin B-type proteins interact with the CDK1 during the G_2_ phase, creating an active complex important for the orderly progression of cell division after DNA synthesis (Draetta *et al*, 1989; Murray *et al*, 1989; Pines and Hunter, 1990). Activation of CDK1 only occurs when sufficient cyclin B1 protein has been synthesized (Solomon *et al*, 1990) and the synthesis of cyclin B1 protein, as for other cyclins, correlates with mRNA accumulation.

**Figure 5:**
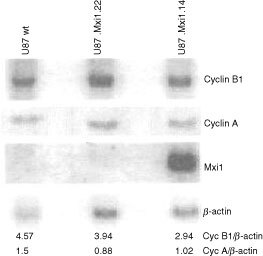
Mxi1 inhibits expression of the *cyclin B1* gene. Northern blot analysis was performed on total RNA extracted from wild-type, Mxi1.22, and Mxi1.14 U87 cell lines. RNAs were size-fractionated on a 1% agarose gel, blotted on a nylon membrane, hybridized with ^32^P-labelled *cyclin B1, cyclin A* and *MXI1* cDNAs and assessed by autoradiography. Exposure was for 3 h (after overnight exposure the endogenous *MXI1* mRNA was visible). To normalize RNA loading, the membrane was re-hybridized with the ^32^P-labelled cDNA of the housekeeping gene β-*actin*. The relative intensity of the bands was quantitated by densitometry.

**Figure 6:**
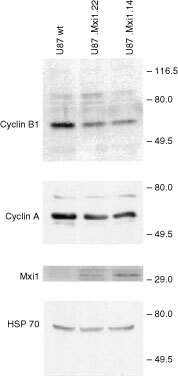
Cyclin B1 is decreased in Mxi1 transfected U87 cells. Protein extracts from cell lines indicated above each lane were blotted and analyzed by anti cyclin B1, cyclin A, Mxi1, and Hsp70 antibodies. Blotted proteins were in similar amounts, as evaluated by Ponceau staining (not shown).

To dissect the molecular mechanism through which Mxi1 induces accumulation in the G_2_/M phase of the cell cycle, we investigated, in U87 proliferating cells, the expression of *cyclin B1* gene and of *cyclin A* as control. The level of the two cyclin mRNAs was evaluated by Northern blot on RNA of U87.Mxi1.14, U87.Mxi1.22 and U87 wild-type cells. *MXI1* and *beta-actin* transcripts were also analyzed on the same membrane. Figure 5

shows that the amount of *cyclin B1* transcript is decreased in U87.Mxi1.14 cells and is similar to wild-type cells in U87.Mxi1.22 cells, while the amount of *cyclin A* transcript is similar in the three cell population. As evaluated by densitometric analysis, the amount of *cyclin B1* transcript is 64% than that of wild-type cells in U87.Mxi1.14 and 86% in U87.Mxi1.22 cells. In agreement with this, immunoblotting analysis of these three cell populations showed that the amount of cyclin B1 protein is decreased in clones 14 and 22 (Figure 6

). These data demonstrate that high levels of Mxi1 prevent the accumulation of *cyclin B1* mRNA in proliferating U87 cells, suggesting that the cell cycle perturbation induced by Mxi1 over-expression depends on the down-regulation of cyclin B1 expression.

### Transcriptional mechanisms are involved in the inhibition of cyclin B1 expression mediated by Mxi1

**Figure 7:**
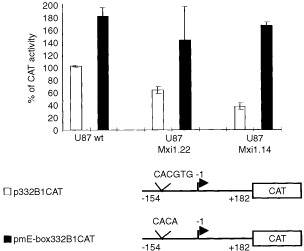
Mxi1 inhibits the promoter activity of the *cyclin B1* gene. *Cyclin B1* promoter CAT-reporter constructs p332B1CAT and pmE-box332 B1CAT (5 μg each) were co-transfected by calcium phosphate in wild-type, Mxi1.22, and Mxi1.14 U87 cell lines along with the CMV-βgal reporter construct (0.5 μg). CAT assay was performed as described (Graham and van der Eb *et al*., 1973). The values, normalized against β-galactosidase activity, are expressed as percentages of the basal activity (100%) assessed by transfecting p332B1CAT into wild-type U87. Results represent the mean of three independent experiments each performed in duplicate.

Human *cyclin B1* mRNA appears at the end of the S phase and its expression peaks during the G_2_ phase of the cell cycle (Pines and Hunter, 1989; Piaggio *et al*, 1995). The transcriptional level of regulation is involved in the induction of expression at the end of the S phase (Cogswell *et al*, 1995; Hwang *et al*, 1995; Piaggio *et al*, 1995; Katula *et al*, 1997). It has been previously demonstrated that the Upstream Stimulatory Factor (USF), binding the E-box sequence in the promoter of the *cyclin B1* gene, is responsible for transcription induction of this gene at the end of the S phase (Cogswell *et al*, 1995). The same E-box also plays a crucial role as a quiescence responsive element in serum-starved NIH3T3 cells (Farina *et al*, 1996). We also found that the over-expression of Max protein in proliferating cells leads to down-regulation of the cyclin B1 protein by interacting with the CACGTG E-box located at position −124/−119 in the promoter of the *cyclin B1* gene (Farina *et al*, 1996). Based on these findings we asked whether Mxi1 could modulate the expression of the endogenous *cyclin B1* gene directly through the transcriptional inhibition of the *cyclin B1* promoter. To answer this question, we transiently transfected U87, U87.Mxi1.22, and U87.Mxi1.14 cells, with plasmids carrying the CAT reporter gene under the control of a *cyclin B1* wild-type promoter fragment (p332B1CAT) or a promoter fragment carrying a mutated E-box (pmE-box332B1CAT). The activity of p332B1CAT in U87 wild-type cells was made 100%. As shown in Figure 7

, the activity of p332B1CAT decreases to 63% in U87.Mxi1.22 cells and to 36% in U87.Mxi1.14 cells. In contrast, CAT activity of pmE-box332B1CAT does not decrease in Mxi1 over-expressing cells and is similar in the three cell lines. Interestingly, in wild-type cells the activity of the pmE-box332B1CAT is higher than that of p332B1CAT, thus indicating that a negative control of the *cyclin B1* promoter activity is lost, at least in part, in the absence of a functional E-box, suggesting a role for endogenous Mxi1. Taken together these results demonstrate that the Mxi1 over-expression in glioblastoma cells inhibits *cyclin B1* promoter activity through the E-box in a dose-dependent fashion, suggesting that Mxi1 could directly modulate the expression of the endogenous *cyclin B1* gene through transcriptional inhibition.

### Max/Mxi1 heteridimers bind the E-box present on the cyclin B1 promoter

**Figure 8:**
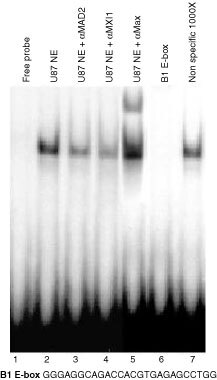
Max/Mxi1 heterodimers bind the E-box present on the *cyclin B1* promoter. Gel mobility retardation assays were performed with a double-stranded, ^32^P-labelled oligonucleotide, spanning position −133/−110, containing the E-box of the *cyclin B1* promoter (B1 E-box). Addition of nuclear extract from U87 cells resulted in a retarded band. A 200-fold molar excess of unlabelled probe (lane 6) but not an oligonucleotide containing a non-specific binding site (lane 7) specifically inhibits the formation of the complex. In lanes 3, 4, nuclear extracts were pre-incubated with anti-Mxi1 antibodies and, in lane 5, with anti-Max antibody.

We previously demonstrated that the Max protein recognizes the E-box present on the *cyclin B1* promoter (Farina *et al*, 1996). By EMSAs we verified the ability of Mxi1 to bind the cyclin B1 E-box. Electromobility shift assays, performed with U87 nuclear extracts, reveal one protein complex binding to the radiolabelled probe, spanning nt −133 to −110 in the *cyclin B1* promoter (B1 E-box) (Figure 8

). A 200-fold molar excess of unlabelled probe (Figure 8, lane 6) specifically inhibits the complex, but not a 1000-fold molar excess of an unrelated probe B1upCCAAT (Farina *et al*, 1999) containing a CCAAT box sequence (lane 7). Direct evidence of Mxi1 binding was obtained through the use of specific anti-Mxi1 antibodies in the binding reactions. U87 nuclear extracts were pre-incubated with two different antibodies against the Mxi1 protein. As shown in Figure 8, lanes 3 and 4 both Mxi1 antibodies interfere, at least in part, with the formation of the complex. As expected an anti-Max antibody modifies the mobility of the complex (Figure 8, lane 5). In particular, 2 μg of antibodies against the Mxi1 protein are necessary to interfere with the formation of the complex, while only 100 ng of the anti-Max antibody are sufficient to modify the mobility of the complex. This apparent discrepancy could be explained with a different affinity of the antibodies towards two different proteins and towards different functional domain of the proteins. Indeed the antibodies against Mxi1 interfere with the DNA binding, thus indicating that they bind the DNA binding domain of Mxi1 and competition for the binding of the probe. Instead, the anti-Max antibody modifies the mobility of the complex without competition with the binding of Mxi1 to the probe. Neither anti-Mxi1 nor anti-Max antibodies completely interfere with the formation or retard the mobility of the complex. It has been previously demonstrated that USF binds this E-box (Cogswell *et al*, 1995; Farina *et al*, 1996), thus the residual molecular complex still occurring in the presence of anti-Mxi1 and anti-Max antibodies could be due to the ability of USF to bind this E-box. Altogether, these results provide evidence that, at least *in vitro*, Mxi1 recognizes the E-box present on the *cyclin B1* promoter.

## DISCUSSION

In the present study we describe one molecular mechanism by which Mxi1 can act as a tumour suppressor in glioblastomas. First, we demonstrate that Mxi1 over-expression causes an inhibition of proliferation of U87 glioblastoma cells *in vitro* due to an accumulation of the cells in the G_2_/M phase of the cell cycle. A similar finding was reported by another group (Wechsler *et al*, 1997). Furthermore, we also demonstrate that Mxi1 over-expression inhibits tumorigenesis of U87 cells in nude mice. This result is in agreement with those coming from *MXI1*-deficient mice that show increased susceptibility to tumorigenesis (Schreiber-Agus *et al*, 1998). The investigation of the *mxi1* coding sequence in primary glioblastomas does not identify this gene as a major target on chromosome 10q. Indeed SSCP analysis of 36 tumour DNA failed to identify mutations (data not shown).

These data imply that the mechanism through which Mxi1 exerts its tumour suppressor function could be of general interest. We demonstrate here that this mechanism includes the loss of cyclin B1 accumulation through the inhibition of *cyclin B1* promoter activity and the possible consequent accumulation of the cells in the G_2_/M phases of the cell cycle. This G_2_/M accumulation is of particular interest, considering that over-expression in malignant gliomas of the other Max interactor, Mad1, induces G_1_/S arrest without obvious perturbations of the G_2_/M progression (Roussel *et al*, 1996). This difference and the observation that Mad1 is highly expressed only in non proliferating, post-mitotic cells while Mxi1 is present in cycling cells (Hurlin *et al*, 1995), suggests that these two proteins affect cell cycle progression at different phases.

In this study we demonstrate that Mxi1 binds the E-box present on the *cyclin B1* promoter *in vitro* and that high levels of Mxi1 inhibit the *cyclin B1* promoter activity in an E-box dependent manner *in vivo*. These results raise the hypothesis that an excess of Mxi1 protein produces an excess of Mxi1/Max heterodimers that may compete with USF for the binding to the E-box of *cyclin B1* promoter (Cogswell *et al*, 1995; Farina *et al*, 1996). Also in this case the mechanism of action of Mxi1 is different from that of Mad1, since previous results demonstrated that the inhibitory effect of Mad1 on the *cyclin B1* promoter is E-box independent (Farina *et al*, 1996). This diversity confirms that Mxi1 and Mad1 may act during the cell cycle in pathways involving different molecular mechanisms.

In conclusion, we have reported that the molecular mechanism through which Mxi1 can act as an inhibitor of proliferation and tumorigenesis of U87 glioblastoma cells includes the inhibition of *cyclin B1* promoter activity through the E-box and the possible consequent loss of cyclin B1 accumulation. Altogether, our results indicate that Mxi1 mediates the down-regulation of *cyclin B1* gene expression in malignant gliomas suggesting that this gene is a functional target of the tumour suppressor activity of Mxi1.
